# *Cryptococcus gattii* Risk for Tourists Visiting Vancouver Island, Canada

**DOI:** 10.3201/eid1301.060945

**Published:** 2007-01

**Authors:** Jens Lindberg, Ferry Hagen, Alex Laursen, Jørgen Stenderup, Teun Boekhout

**Affiliations:** * Skejby Hospital, Aarhus, Denmark; † CBS Fungal Biodiversity Center, Utrecht, the Netherlands; ‡ Herning Hospital, Herning, Denmark; § University Medical Centre, Utrecht, the Netherlands

**Keywords:** Cryptococcus gattii, tourism, Vancouver Island, British Columbia, Canada, letter

**To the Editor:** An unprecedented outbreak of *Cryptococcus* *gattii* genotype amplified fragment length polymorphism (AFLP)6/VGII on Vancouver Island, British Columbia, Canada, is affecting both human and animal hosts with normal immunity (*1*–*3*). So far, >100 human cases, including at least 6 fatalities, have been reported by the British Columbia Centre for Disease Control (*4*), (www.bccdc.org, www.cbc.ca). Vancouver Island is a major tourist destination, with ≈7.5 million visits each year (www.bcstats.gov.bc.ca). We report the first known intercontinental transmission of *C. gattii* from this outbreak in a tourist from Denmark who visited Vancouver Island. This case indicates a potential risk for tourism-related acquisition.

A 51-year-old, HIV-negative, apparently immunocompetent man from the Netherlands, with known psoriatic gout and under treatment with a nonsteroidial antiinflammatory drug, was admitted to a hospital in Herning, Denmark, with chest pain radiating to the left shoulder and arm, lasting for 1 day. Six weeks before his admission, he returned to Denmark from a 3-week trip to Canada, during which he visited Victoria and surrounding areas on the eastern coast of Vancouver Island for 7 days. During their stay, the patient and his 3 fellow travelers visited gardens and studied the local natural vegetation.

During his stay in Canada, the patient had no symptoms, and symptoms had not developed in any of his family members as of October 2006. On admission to the hospital, his temperature was 38.2°C, and a chest radiograph showed 3 large nodular infiltrates suspect for malignancy or abscesses. Neither bacterial nor fungal pathogens could be isolated from sputum by classic and molecular methods. After 4–5 days, his temperature was 40°C, a productive cough with dyspnea was noted, and his condition deteriorated. A chest radiograph showed progression of the infiltrates, and a computed tomography scan of the abdomen and chest showed infiltrates near the pleura, suggesting encapsulated fluid (Figure). An ultrasound-guided lung biopsy was performed, and mucoid material was aspirated. Microscopy and a culture from the aspirate showed a cryptococcal isolate. This isolate was further identified by internal transcribed spacer and D1/D2 sequencing, as well as amplified fragment-length polymorphism analysis (*2*). In addition, detailed genotyping was performed by using sequences of 7 genes (IGS*, CAP10*, *GPD1*, *LAC1*, *MPD1*, *PLB1*, and *TEF1*; GenBank accession nos. DQ861593–DQ861599) (*5*).

**Figure:**
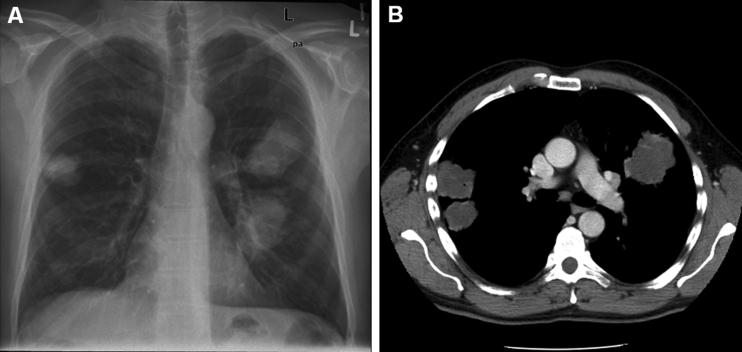
A) Chest radiograph and B) computed tomography scan of the patient showing 3 nodular *Cryptococcus gattii* infiltrates near pleura.

Extensive molecular research showed that this isolate belonged to the highly virulent AFLP genotype 6A (VGIIa) of *Cryptococcus gattii*, which is the major genotype involved in the Vancouver Island *C. gattii* outbreak (*1*–*4*). All 7 sequenced genes had a complete match with the sequence types specific for isolates involved in the Vancouver Island outbreak (*5*). Thus, we conclude that the pathogen was acquired during the patient’s visit to Vancouver Island and imported to Denmark. The presence of 3 cryptococcal masses of more or less equal size suggests that the patient was exposed to a high concentration of infectious cells of *C. gattii*. The observed incubation time of 6 weeks is shorter than that was previously reported for infections related to the Vancouver Island outbreak (2–11 mo) (*4*). These observations, in combination with the absence of any known predisposing factor in this patient, such as smoking or treatment with corticosteroids, suggest that this specific AFLP6 genotype of *C. gattii* is highly virulent (*4*,*5*).

This case suggests a potential risk of tourists acquiring cryptococcosis while visiting Vancouver Island. Therefore, we recommend tourists and medical staff of healthcare centers worldwide be alert for symptoms of cryptococcosis after travel to Vancouver Island.
